# Letter to the Editor: Use of some inappropriate terms in Spanish in Oral Medicine and Pathology

**DOI:** 10.4317/medoral.21158

**Published:** 2016-01-31

**Authors:** José-Manuel Aguirre-Urizar, Adalberto Mosqueda-Taylor

**Affiliations:** 1 Departamento de Estomatología II. UFI 11/25. Universidad del País Vasco/EHU. Leioa. España; 2 Departamento de Atención a la Salud. Universidad Autónoma Metropolitana. Xochimilco. México D.F. México

Dear Editor: According to the Dictionary of the Spanish Language of the Royal Spanish Academy (DLERAE) (1) anglicism refers to those words or terms of English language that are used in another. The Spanish language has acquired and uses numerous anglicisms, especially for words that have no option to be translated into Spanish, such as those absent in this language like *internet, wifi, whisky*, etc. In Medicine a lot of anglicisms are inevitably used today in the medical language, such as: *stress, test, distress, gold standard, score, shunt, level*, etc. Dentistry also commonly uses many anglicisms, such as *forceps, bonding, inlays, composite*, etc. that would be justified in most cases due to the absence of a fully equivalent word in Spanish. However, in some instances this is debatable, and their use only represents a linguistic simplification. Our intention by sending this letter is to draw attention of the readers of this journal over temptation of giving a Spanish style to certain English words employed in Oral Medicine and Pathology. To illustrate this we present three examples of this inadequate circumstance. The first refers to “*disorder*”, a term widely used in recent years in connection with oral precancer (oral potentially malignant disorders). This word is usually translated as “*desorden*”, a word which although may sound very similar in Spanish, does not means exactly the same and it is not justified to use it. According to the DLERAE (1) when we say “*desorden*” we mean: 1) confusion and disorderly or 2) disturbance of order and discipline of a group, a meeting, a community of people, or 3) disturbance that alters the tranquility of public, or 4) excess or abuse. Therefore, we consider more appropriate to translate it as “*trastorno*”, whose meanings in DLERAE (1) are: 1) action and effect of modify the permanent features of something or the development of a process, and 2) mild impairment of health. The word “*trastornar*” has, among others, the meanings: 1) reverse the regular order of something and 2) to alter the normal functioning of something or the activity of someone. Our proposal is to translate “oral potentially malignant disorders” into Spanish as “trastornos orales potencialmente malignos”. Similarly, the term “*verrucous*”, which defines a similar clinical appearance of verrucae is very often mistakenly translated into Spanish as “*verrucoso*”, a word that has no basis in the DLERAE (1). We therefore believe that this word should be translated as “*verrugoso*”, and therefore “verrucous leukoplakia” should be translated as “leucoplasia verrugosa”, analogous to the translation of “verruga” for the English term “verruca”. Finally, another inadequate translation into Spanish occurs with the word “*necrotizing*”, which is usually translated incorrectly as “*necrotizante*”, a term that does not exist in the DLERAE (1). Thus, we believe it should be translated into Spanish as “*necrosante*” coming from the verb “*necrosar*” (action to produce necrosis)1, so that “necrotizing sialometaplasia” would be translated as “sialometaplasia necrosante” as “necrotizing granulomas” would be “granulomas necrosantes”. The confusion stems from the act of trying to translate the English verbs ending with “tize” as “tizar”, when these should be left in their Spanish infinitive form “ar”, and therefore “anesthetize” is “anestesiar” or “phagocytize” is “fagocitar”, etc.
